# Targeting uridine–cytidine kinase 2 induced cell cycle arrest through dual mechanism and could improve the immune response of hepatocellular carcinoma

**DOI:** 10.1186/s11658-022-00403-y

**Published:** 2022-11-26

**Authors:** Dehai Wu, Congyi Zhang, Guanqun Liao, Kaiming Leng, Bowen Dong, Yang Yu, Huilin Tai, Lining Huang, Feng Luo, Bin Zhang, Tiexiang Zhan, Qiuhui Hu, Sheng Tai

**Affiliations:** 1 Department of Hepatic Surgery, Second Affiliated Hospital of Harbin Medical University, #246Xuefu Road, Harbin, 150086 Heilongjiang China; 2 Department of Hepatobiliary Surgery, Foshan Hospital Affiliated to Southern Medical University, Foshan, 528000 China; 3 Department of Hepatobiliary Surgery, Qingdao Municipal Hospital, Qingdao, 266071 China; 4 Department of Biochemistry & Molecular Biology, Harbin Medical University, Harbin, 150081 China; 5 McGill Mathematics and Statistics Department, Montreal, Canada; 6 Department of Hepatobiliary Surgery, Suzhou Municipal Hospital, Gusu School, Nanjing Medical University, Suzhou, 215008 China; 7 Department of Intensive Care Unit, Seventh Affiliated Hospital of Sun Yat-Sen University, Shenzhen, 528406 China; 8 Department of Hepatobiliary Surgery, Second Cancer Hospital of Heilongjiang Province, Harbin, 150088 China

**Keywords:** Uridine–cytidine kinase 2, Hepatocellular carcinoma, Microenvironment, Secretory phenotype, Pyrimidine metabolism

## Abstract

**Background:**

Pyrimidine metabolism is critical for tumour progression. Uridine–cytidine kinase 2 (UCK2), a key regulator of pyrimidine metabolism, is elevated during hepatocellular carcinoma (HCC) development and exhibits carcinogenic effects. However, the key mechanism of UCK2 promoting HCC and the therapeutic value of UCK2 are still undefined. The aim of this study is to investigate the potential of UCK2 as a therapeutic target for HCC.

**Methods:**

Gene expression matrices were obtained from public databases. RNA-seq, co-immunoprecipitation and RNA-binding protein immunoprecipitation were used to determine the mechanism of UCK2 promoting HCC. Immune cell infiltration level and immune-related functional scores were evaluated to assess the link between tumour microenvironment and UCK2.

**Results:**

In HCC, the expression of UCK2 was upregulated in part by TGFβ1 stimulation. UCK2 promoted cell cycle progression of HCC by preventing the degradation of mTOR protein and maintaining the stability of PDPK1 mRNA. We also identified UCK2 as a novel RNA-binding protein. Downregulation of UCK2 induced cell cycle arrest and activated the TNFα/NFκB signalling pathway-related senescence-associated secretory phenotype to modify the tumour microenvironment. Additionally, UCK2 was a biomarker of the immunosuppressive microenvironment. Downregulated UCK2 induced a secretory phenotype, which could improve the microenvironment, and decreased UCK2 remodelling metabolism could lower the resistance of tumour cells to T-cell-mediated killing.

**Conclusions:**

Targeting UCK2 inhibits HCC progression and could improve the response to immunotherapy in patients with HCC. Our study suggests that UCK2 could be an ideal target for HCC.

**Supplementary Information:**

The online version contains supplementary material available at 10.1186/s11658-022-00403-y.

## Background

Hepatocellular carcinoma (HCC) is the fourth leading cause of cancer-related mortality, and its incidence is increasing worldwide [1]. The most common causes of HCC are chronic hepatitis B virus and hepatitis C virus infection [2]. On the basis of proteomic profiling, patients with HCC can be divided into three subgroups that show distinct molecular features in metabolic reprogramming, microenvironment dysregulation and cell proliferation [3]. A previous study demonstrated that dysregulation of metabolic genes promotes HCC proliferation by regulating the cell cycle, suggesting the existence of a regulatory network [4].

Pyrimidine metabolism is critical for DNA replication, RNA synthesis and cellular bioenergetics as well as for cancer cells to maintain uncontrolled tumour growth by continuously supplying dNTPs [5, 6]. In addition, pyrimidine metabolites can induce epithelial to mesenchymal transition to promote tumour metastasis, playing a non-proliferative role in pyrimidine metabolism in cancer [7]. Uridine–cytidine kinase 2 (UCK2), a key regulator of pyrimidine metabolism, catalyses the phosphorylation of uridine and cytidine to form uridine monophosphate and cytidine monophosphate [8]. UCK2 is frequently upregulated in various tumour types and serves as an indicator of poor prognosis [9–11]. High levels of UCK2 promote cancer cell proliferation and metastasis by activating the Wnt/β-catenin and EGFR–AKT signalling pathways [12, 13].

Previous studies have shown the oncogenic role of UCK2 in the progression of HCC. However, the link between UCK2 and tumour microenvironment has not been investigated. In the present study, we found that knockdown of UCK2 induced cell cycle arrest through dual mechanisms in HCC and that targeting UCK2 promoted the secretory features involved in the senescence-associated secretory phenotype (SASP) and inflammasomes. Senescence is a cellular state in cells undergoing cell cycle arrest; these cells can remain metabolically active, resulting in the secretion of pro-inflammatory factors to result in SASP [14]. Both SASP and inflammasomes exhibit tumour-promoting and tumour-suppressive roles, which is determined by the cell type, tissue of origin and primary cellular stressor [14–16]. Secretory factors, such as interleukin 1A (IL1A), IL1B, IL18, CXCL10, tumour necrosis factor α (TNFα) and intracellular adhesion molecule 1 (ICAM1), are involved in natural killer (NK) cell recruitment, proliferation and activation [17]. In addition, downregulation of UCK2 increases the expression of major histocompatibility complex (MHC) I, which sensitizes cancer cells to T-cell-dependent killing [18]. In the present study, we aimed to explore the mechanism of UCK2 promoting HCC and the potential therapeutic value.

## Methods

### Clinical specimens and cell lines

The Cancer Genome Atlas Liver Hepatocellular Carcinoma (TCGA-LIHC), Gene Expression Omnibus and International Cancer Genome Consortium datasets were acquired from the SangerBox platform (http://vip.sangerbox.com/). Tissue microarray chips (164 tissue dots, including 78 tumour dots for further analysis) were obtained from Shanghai Outdo Biotech Company (Shanghai, China). The ethics committee of the Second Affiliated Hospital of Harbin Medical University approved this study protocol. HCCLM3 (CTCC-400-0193) and Hep3B (CTCC-001-0021) cell lines were purchased from MeisenCTCC. The cell lines were cultured in Dulbecco’s modified Eagle medium (DMEM) supplemented with 10% foetal bovine serum (FBS) and 1% antibiotics (100 U/mL penicillin and 100 µg/mL streptomycin). All cell lines were cultured at 37 °C in a 5% CO_2_ incubator.

### Lentivirus, stable cell line construction and quantitative real-time PCR

The lentiviral vector system and empty vector were purchased from GeneChem Corporation (Shanghai, China). Stable cell lines expressing the target gene or negative control were selected by adding 0.5 μg/mL puromycin into the medium. Total RNA was extracted and quantified according to the protocol of the RNA purification kit (Thermo Fisher Scientific, Waltham, MA, USA). Quantitative real-time PCR was performed using SYBR Green Power Master Mix (Promega, Madison, WI, USA). The following primers were used for quantitative reverse-transcription PCR: UCK2 forward: 5′-GCCCTTCCTTATAGGCGTCAG-3′; UCK2 reverse: 5′-CTTCTGGCGATAGTCCACCTC-3′; GAPDH forward: 5′-AGAAGGCTGGGGCTCATTTG-3′, GAPDH reverse: 5′-AGGGGCCATCCACAGTCTTC-3′.

### HCC stem cell detection, cell proliferation and cell cycle assay

To evaluate HCC stem cell characteristics, 1000 cells were seeded into 24-well plates pre-treated with the CSwell 600 kit (Suzhou Jiyan Biotech Co., Ltd., Jinang City, China). After 24 h, the number of cell spheres was counted under a microscope. To examine cell proliferation, the cells were seeded into 96-well plates at 2 × 10^3^ cells per well, and cell viability was measured using Cell Counting Kit-8 assays (Dojindo Molecular Technologies, Kumamoto, Japan) at different timepoints. For cell cycle tests, 4 × 10^4^ cells were stained according to the protocol of the Cycle TESTTM PLUS DNA Reagent Kit (BD Biosciences, Franklin Lakes, NJ, USA) and analysed using flow cytometry (FC500, Beckman Coulter, Brea, CA, USA).

### Transwell assay

The migration and invasion of HCC cells were evaluated in Transwell assays. Chambers with or without pre-coated Matrigel were placed in a 24-well plate. DMEM supplemented with 10% FBS was added to the lower chamber. Cells (5 × 10^4^) suspended in 100 µL of DMEM without FBS were seeded into the upper chamber and cultured for 24 h.

### Western blotting and co-immunoprecipitation (co-IP)

Cells were lysed in radioimmunoprecipitation assay buffer containing protease and phosphatase inhibitors. The proteins were separated by sodium dodecyl sulphate polyacrylamide gel electrophoresis and transferred onto polyvinylidene fluoride membranes. After blocking with 5% skimmed milk at room temperature for 1 h, the membranes were incubated with primary antibodies overnight. The primary and secondary antibodies are listed in Additional file 5: Table S1. The membranes were then incubated with secondary antibodies for 1 h at room temperature, and the bands were detected using an enhanced chemiluminescence kit. For co-immunoprecipitation, the cells were incubated with pre-cold immunoprecipitation (IP) lysis buffer for 5 min and then transferred into a 1.5 mL tube for centrifugation at 13,000*g* for 10 min. The supernatant was collected, and the IP process was performed according to the protocol of the A Dynabeads protein G IP Kit (Thermo Fisher Scientific). Western blotting was performed as described above.

### Immunohistochemical (IHC) staining

Deparaffinized tissue sections were stained with diaminobenzidine (DAB kit; Vector Laboratories, Burlingame, CA, USA) and counterstained with haematoxylin (Sigma, St. Louis, MO, USA) to visualize the immunoreaction product following the manufacturer’s protocol. The staining intensity was scored as 0 (negative), 1 (weak), 2 (moderate) and 3 (strong). The percentage scores were defined as 0, < 5%; 1, 5–25%; 2, 26–50%; 3, 51–75%; 4, > 75%. The histological score for each section was calculated using the following formula:

$${\text{Histological score}} = {\text{proportion score}} \times {\text{intensity score}}.$$

### Immunofluorescence (IF) assays

The cells were seeded onto coverslips in six-well plates and incubated for 24 h. The cells were fixed with 4% paraformaldehyde and penetrated with 0.1% Triton-X-100. After incubation with the primary antibody for ICAM1 (ab222736, ABCAM), secondary antibody (Invitrogen, Carlsbad, CA, USA), and DAPI (Vector Laboratories) in sequence, images of the cells were acquired using a DMRA fluorescence microscope (20×, Olympus, Tokyo, Japan).

### RNA-binding protein immunoprecipitation (RIP)

The cells were lysed using RIP assay buffer. The cell lysates were incubated with beads that had been pre-coated overnight with an antibody for UCK2 (ab241281, ABCAM). RNA was extracted from the samples according to the protocol of the RIP kit (Millipore, Billerica, MA, USA). Quality control and RIP sequencing were performed by GeneChem Corporation.

### Determination of immune cell infiltration level and immune-related functional scores

Information on pan-cancer immune cell infiltration was obtained from TISIDB, an integrated repository portal for tumour–immune system interactions [19]. Using the CIBFERSORT database, we identified 22 immune cell types. The immune score, stromal score and tumour purity were generated using the R package “ESTIMATE”. Tumour immune dysfunction and exclusion (TIDE) and immunophenoscore analyses were performed as previously described [20, 21].

### Statistical analysis

The correlation of gene expression and the survival of patients with HCC was determined using Lasso–Cox analysis with R package “glmnet”. Student’s *t*-test was used for comparisons between the two groups. Pearson’s correlation analysis was used to determine the linear relationship between the two groups. Multivariate analysis was performed using the Cox regression model. Kaplan–Meier curves were used to compare survival, and the log-rank test was used to compare survival between different groups. The results were considered as significant when *p* < 0.05.

## Results

### UCK2 was upregulated in HCC and involved in tumour stemness

**Fig. 1:**
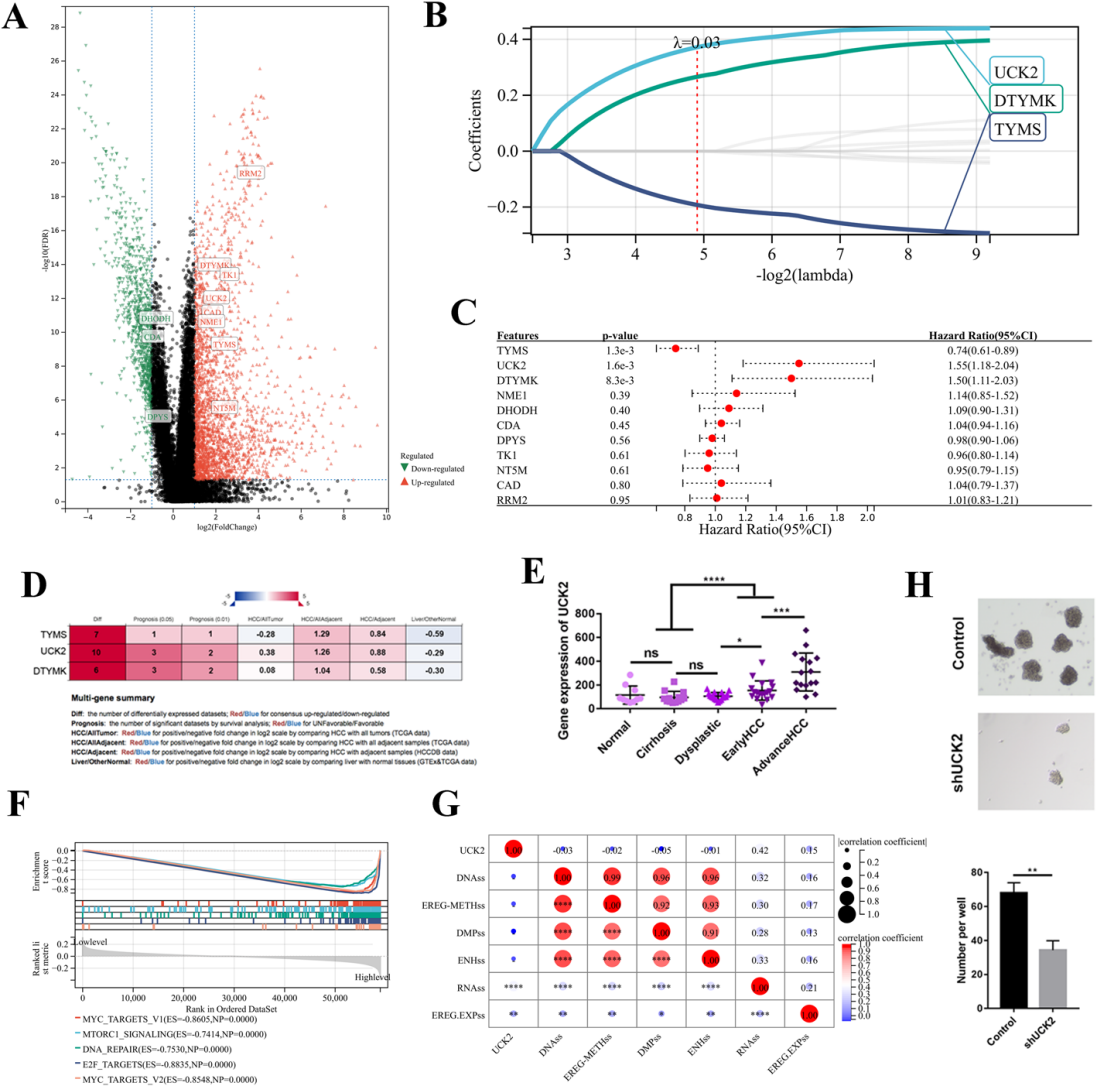
UCK2 was upregulated in HCC and involved in tumour stemness. **A** Volcano plot for different expression genes between tumour and adjacent tissues. **B**, **C** Lasso–Cox regression analysis filtered genes significantly associated with the prognosis of HCC. **D** Integrative molecular database of HCC (HCCDB) database was used to examine the role of TYMS, UCK2 and DTYMK in HCC. **E** The expression of UCK2 in different stage during HCC development, data derived from GEO6764. **F** GSEA analysis revealed the signalling pathways related to UCK2. **G** Correlation of UCK2 and stemness features. **H** 3D sphere formation for the indicated cell lines, *p < 0.05; **p < 0.01; ***p < 0.001

Pyrimidine metabolism is critical for tumour proliferation. To identify an ideal target involved in pyrimidine metabolism for treating HCC, we compared gene expression between 50 pairs of HCC and adjacent tissues. Gene expression data were obtained from TCGA-LIHC dataset. Eleven genes related to pyrimidine metabolism were significantly dysregulated during HCC development (Fig. 1A). Eleven candidates were filtered using Lasso–Cox regression analysis. The results revealed that UCK2, DTYMK and TYMS were independent indicators of HCC prognosis (Fig. 1B, C). TYMS indicated a favourable prognosis, whereas UCK2 and DTYMK indicated unfavourable prognosis (Fig. 1C). Compared with DTYMK, upregulation of UCK2 in HCC was detected in a larger number of individual studies, and UCK2 was more specific for HCC (Fig. 1D, Additional file 1: Fig. S1A). Patients with higher levels of UCK2 predicted to have poor prognosis was also detected in individual studies (Additional file 1: Fig. S1B). UCK2 expression gradually increased during HCC development (Fig. 1E). Gene set enrichment analysis (GSEA) suggested that oncogenic signalling pathways, including the mTORC1, P13K/AKT, Wnt/β-catenin, NOTCH and TGFβ signalling pathways, were enriched in patients with higher levels of UCK2 (Fig. 1F). These pathways are also involved in tumour stemness. A relationship between the gene expression of UCK2 and tumour stemness features was detected on the basis of gene expression data from TCGA-LIHC dataset. Six tumour stemness signatures were constructed, including including RNA expression-based (RNAss), epigenetically regulated RNA expression-based (EREG.EXPss), DNA methylation-based (DNAss), epigenetically regulated DNA methylation-based (EREG-METHss), differentially methylated probes-based (DMPss) and enhancer elements/DNA methylation-based (ENHss), differentially methylated probe-based, and enhancer elements/DNA methylation-based signatures. Gene expression of UCK2 was strongly related to the RNAss signature, suggesting that UCK2 is involved in the transcriptional regulation of tumour stemness (Fig. 1G). Decreased UCK2 significantly inhibited sphere formation of HCCLM3 cells (Fig. 1H). These results demonstrate that upregulated UCK2 is involved in stemness during HCC development.

### UCK2 was elevated by TGFβ signalling pathway

**Fig. 2:**
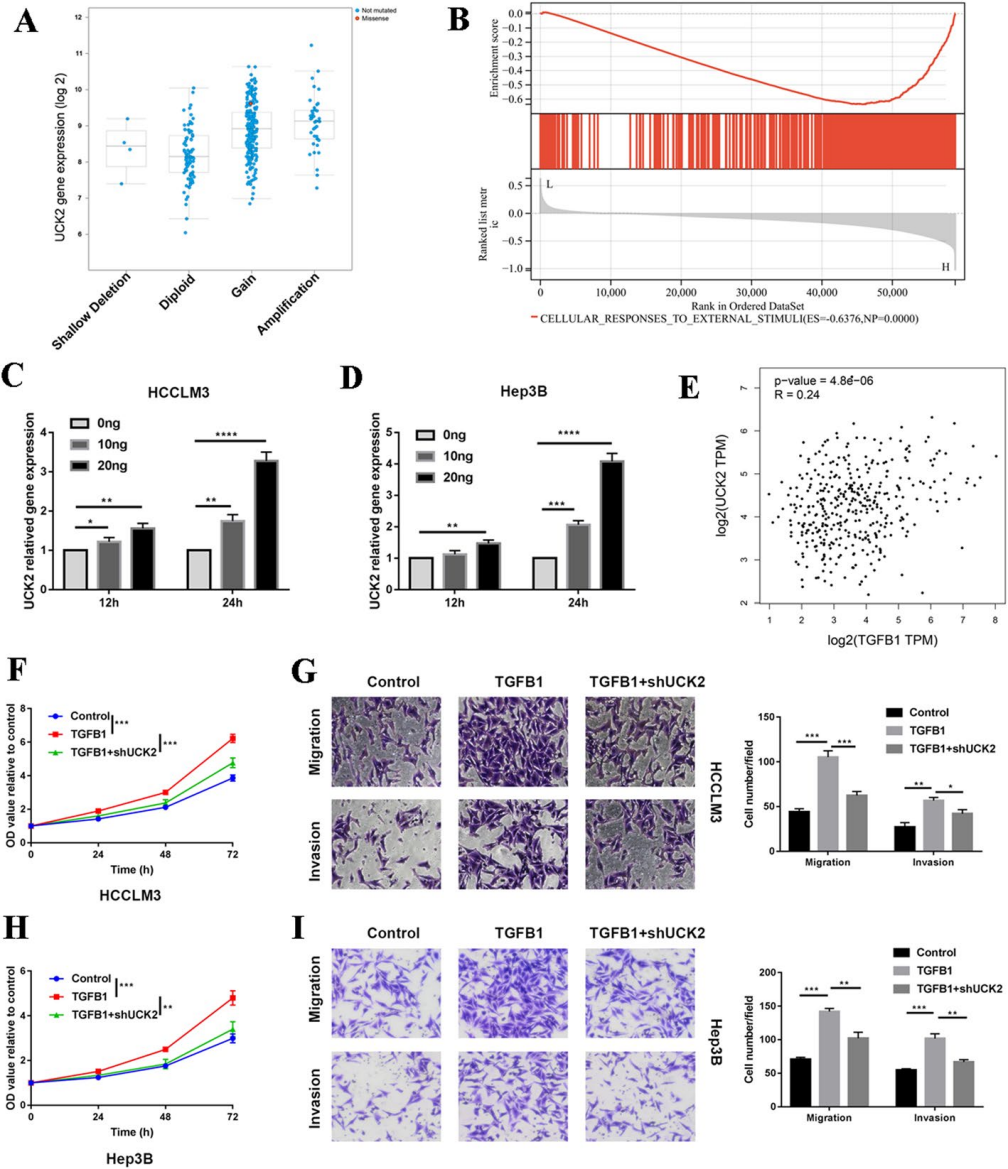
UCK2 was elevated by TGFβ signalling pathway. **A** Gene expression of UCK2 with the copy number variation. **B** GSEA analysis revealed that gene expression of UCK2 was associated with extracellular stimuli. **C**, **D** 10 ng and 20 ng TGFβ1 were added to HCCLM3 and Hep3B cell lines, respectively; the gene expression of UCK2 was detected in different timepoints using qPCR. **E** Correlation between gene expression of UCK2 and TGFβ1. **F**, **H** CCK8 assays was used to determine the proliferation of the indicated cell lines and the value of OD450 was related to control. **G**, **I** The migration and invasion abilities of the indicated cell lines were detected by Transwell assays in the left panel, and the statistical analysis is presented in the right panel. *p < 0.05; **p < 0.01; ***p < 0.001; ****p < 0.0001

However, the mechanism of UCK2 upregulation in cancer remains unclear. A recent study showed that expression of the UCK2 gene in HCC was negatively correlated with its DNA methylation level [8]. In addition, the gene expression of UCK2 was significantly positively correlated with all four DNA methylation transferases in different tumour types (Additional file 2: Fig. S2A). Demethylation by 5-azacytidine-2′-deoxycytidine in the HCCLM3 and Hep3B cell lines increased the expression of UCK2 (Additional file 2: Fig. S2B). These results indicate that UCK2 is regulated by epigenetic modifications in HCC. UCK2 expression was also elevated following copy number amplification, and rare mutations were detected in the UCK2 genome in HCC (Fig. 2A). The GSEA results suggested that upregulation of UCK2 in HCC is associated with extracellular stimuli (Fig. 2B). Here, we found that UCK2 was increased in the HCCLM3 and Hep3B cell lines following TGFβ1 stimulation (Fig. 2C, D). The expression of UCK2 was also positively correlated with that of TGFβ1 in TCGA-LIHC dataset (Fig. 2E). Downregulation of UCK2 attenuated the pro-tumorigenic effect of TGFβ1 (Fig. 2F–I). The knockdown efficiency of UCK2 by lentivirus was detected using quantitative real-time PCR (Additional file 2: Fig. S2C). We found that downregulated UCK2 decreased the expression of SULF2 (Additional file 2: Fig. S2D), which can promote the release of pro-angiogenic factors, including TGFβ1, from the cell surface or extracellular matrix [22]. UCK2 was also positively correlated with regulatory T cells, which are among the major sources of TGFβ1 (Additional file 2: Fig. S2E). These results reveal that UCK2 was upregulated by TGFβ1 and formed a feedback loop via SULF2.

### Overview of UCK2 in human cancers

**Fig. 3:**
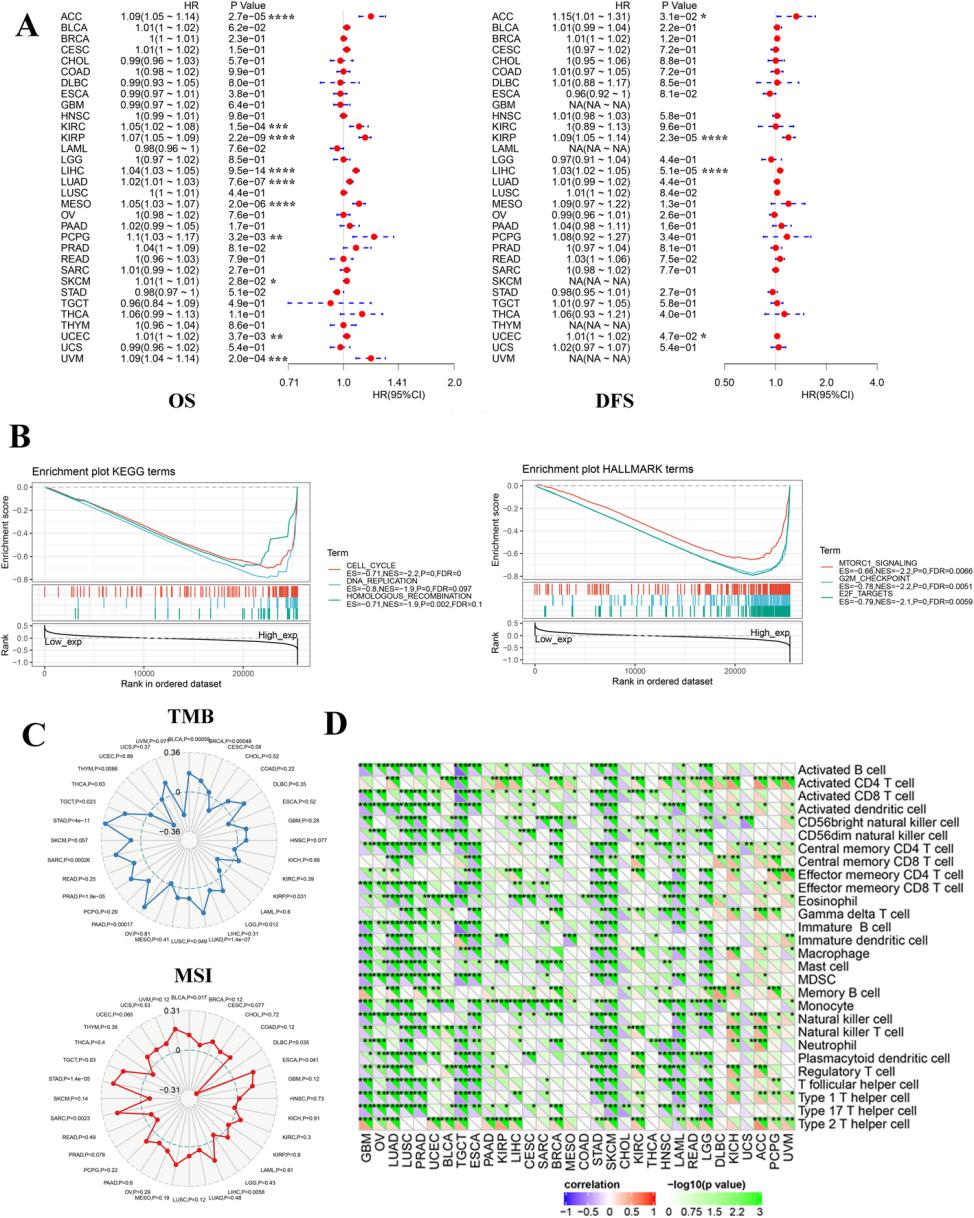
Overview of UCK2 in human cancers. **A** Univariate Cox regression analysis estimating the prognostic value of UCK2 in different cancer types from TCGA database. **B** GSEA analysis revealed the signalling pathways of KEGG and Hallmark terms related to UCK2 in pan-cancer. **C** Correlation of UCK2 with TMB and MSI in different tumour types. **D** The correlation of UCK2 with immune cell infiltrating in different tumour types. *p < 0.05; **p < 0.01; ***p < 0.001; ****p < 0.0001

Considering the results of UCK2-related cancer research, we assessed the expression of UCK2 in 32 tumours, including HCC. The prognostic value of UCK2 expression for overall survival (OS) and disease-free survival (DFS) was validated in TCGA cohort using Cox regression analysis. UCK2 served as a poor prognostic biomarker for both OS and DFS in several tumour types (Fig. 3A). The GSEA results showed that gene sets related to the cell cycle and mTORC1 signalling pathway were enriched in patients with high levels of UCK2, suggesting a role for UCK2 in tumour progression (Fig. 3B). UCK2 was positively correlated with the tumour mutation burden (TMB) and microsatellite instability (MSI), which favoured the infiltration of immune effector cells and anti-tumour immune response (Fig. 3C). However, UCK2 was negatively correlated with the infiltration of immune effector cells in most tumour types (Fig. 3D).

### UCK2 promoted cell cycle progression via regulating the expression of mTOR

**Fig. 4:**
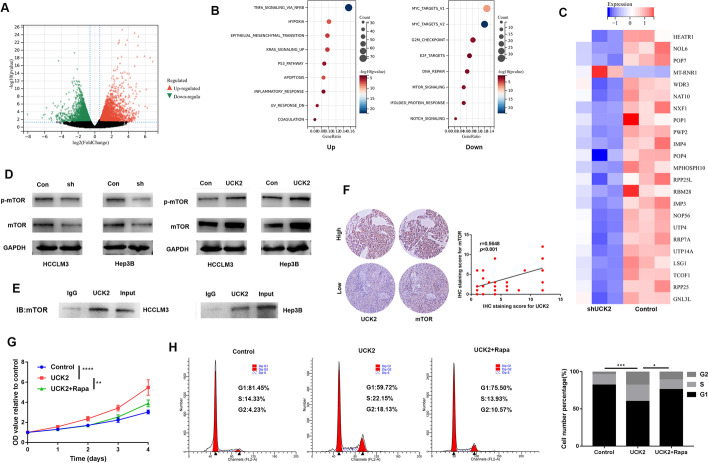
UCK2 promoted cell cycle progression via regulating the expression of mTOR. **A** Volcano plot exhibited the gene expression altered after UCK2 knockdown. **B** The genes upregulated after UCK2 knockdown were enriched in the signalling pathway in the left panel; the genes downregulated are shown in the right panel. **C** Heatmap showed the expression of genes involved in ribosome biogenesis in the indicated cell lines. **D** Western blot (WB) examined the protein expression of p-mTOR and mTOR in the indicated cell lines. **E** The indicated cell lysates were prepared and immunoprecipitated with anti-UCK2 antibody; the protein expression of mTOR was examined by WB. **F** IHC detected the protein expression of UCK2 and mTOR in the left panel, and Pearson’s correlation analysis was used to determine the linear relationship between UCK2 and mTOR in the right panel. **G** CCK8 assays was used to determine the proliferation of the indicated cell lines; cells were treated with 10 μM rapamycin to block the mTOR signalling pathway. **H** Representative images for cell cycle assays in indicated cell lines in the left panel, and statistical analysis in the right panel. *p < 0.05; **p < 0.01; ***p < 0.001; ****p < 0.0001

mRNA sequencing was performed to further explore the role of UCK2 in HCC progression. Differentially expressed genes were filtered and enriched in signalling pathways (Fig. 4A, B). The results showed that downregulation of UCK2 decreased the expression of genes related to the cell cycle and mTOR signalling pathway (Fig. 4B). Knockdown of UCK2 suppressed the expression of genes related to ribosome biogenesis, which is regulated by the mTORC1 signalling pathway (Fig. 4C). Downregulation of UCK2 decreased the expression of both pmTOR and total mTOR (Fig. 4D). The co-immunoprecipitation results showed that UCK2 directly interacted with mTOR (Fig. 4E). Immunohistochemical staining indicated a positive correlation between UCK2 and mTOR protein expression (Fig. 4F). Inhibiting the mTOR signalling pathway by rapamycin attenuated the tumour-promoting effect of UCK2 (Fig. 4G). Upregulation of UCK2 promoted cell cycle progression in HCC, inhibiting the mTOR signalling pathway impeded this effect (Fig. 4H). These data indicate that UCK2 promotes cell cycle progression by regulating the expression of mTOR, which plays a non-metabolic role.

### UCK2 accelerated cell cycle progression by maintaining the stability of PDPK1 mRNA

**Fig. 5:**
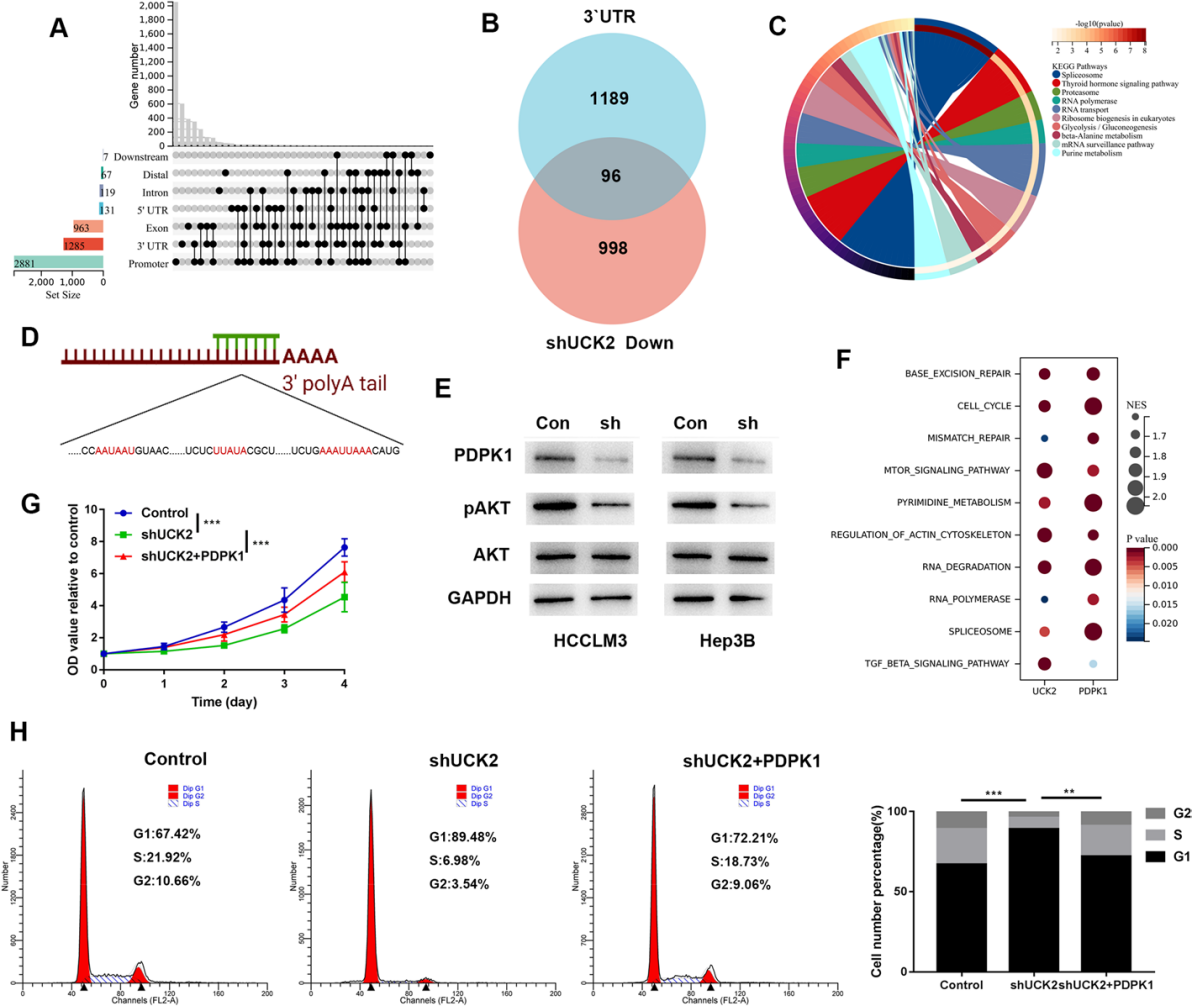
UCK2 accelerated the cell cycle progression via maintain the stable of PDPK1 mRNA. **A** The distribution of peaks on genes. **B** The intersection of genes targeted by UCK2 in 3′UTR and genes downregulated after UCK2 knocking down by shRNA. **C** The signalling pathways that genes of the intersection are enriched in. **D** The AU enriched regions on the UCK2 targeting site. **E** WB examined the protein expression of PDPK1, pAKT and AKT in the indicated cell lines. **F** The common signalling pathways that UCK2 and PDPK1 are related to. **G** CCK8 assays were used to determine the proliferation of the indicated cell lines. **H** Representative images for cell cycle assays in indicated cell lines in the left panel, and statistical analysis in the right panel. *p < 0.05; **p < 0.01; ***p < 0.001; ****p < 0.0001

UCK2 is pivotal in pyrimidine metabolism and activation of cytotoxic nucleoside analogues [23]. Under certain conditions, UCK2 shuttles between the nucleus and cytoplasm [23]. We examined whether UCK2 is involved in genetic information processing. RIP sequencing revealed 9652 peaks related to 4139 genes (Additional file 3: Fig. S3A). The peaks were mostly located in the promoter, 3′UTR, and exon regions (Fig. 5A). These genes are involved in different human disease signalling pathways (Additional file 3: Fig. S3B). UCK2 targeted the 3′UTR regions of 1285 genes and influenced the mRNA stability of 96 genes (Fig. 5A, B). These genes are involved in signalling pathways related to post-transcriptional regulation and metabolism (Fig. 5C). The target sequence of PDPK1 3′UTR by UCK2 (peak over chromosome: Chr16: 2,561,353–2,561,710) contains three AU-rich regions (Fig. 5D). Downregulation of UCK2 decreased the expression of PDPK1 and inhibited the downstream AKT signalling pathway (Fig. 5E). Gene expression of UCK2 was positively correlated with that of PDPK1 (Additional file 3: Fig. S3C). The GSEA results indicated that UCK2 and PDPK1 are involved in several common signalling pathways, including the cell cycle and mTOR signalling pathways (Fig. 5F). Increased expression of PDPK1 attenuated the proliferation suppression and cell cycle arrest induced by knockdown of UCK2 (Fig. 5G, H). Therefore, UCK2 is an RNA-binding protein and promoted cell cycle progression by maintaining the stability of PDPK1 mRNA.

### Targeting UCK2 activated SASP induced by cell cycle arrest

**Fig. 6:**
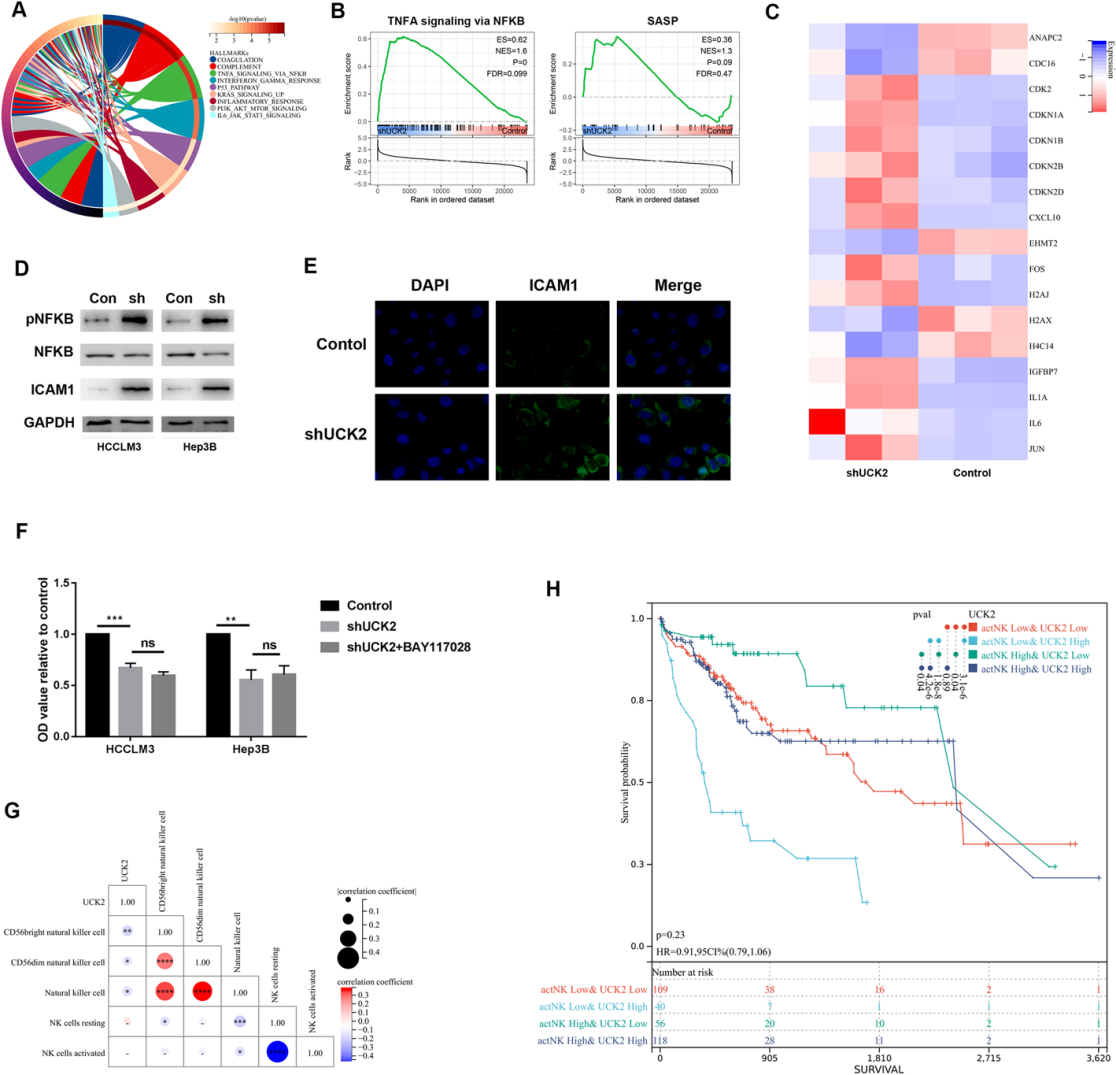
Targeting UCK2 facilitated NK cell surveillance. **A** Genes related to innate immune system were enriched in signalling pathway. **B** GSEA revealed the relationship between signalling pathways and UCK2. **C** Heatmap showed the expression of genes involved in SASP in the indicated cell lines. **D** WB examined the protein expression of pNFKB, NFKB and ICAM1 in the indicated cell lines. **E** IF detected the expression of ICAM1 in the indicated cell lines. **F** CCK8 assays was used to determine the proliferation of the indicated cell lines; the cells were incubated with NFKB signalling pathway inhibitor BAY11-7082, 7.5 µM. **G** Correlation between UCK2 and different types of NK cell. **H** Kaplan–Meier curve suggested the combined effect of UCK2 and activated NK cell on the prognosis of HCC. *p < 0.05; **p < 0.01; ***p < 0.001; ****p < 0.0001

Downregulated UCK2 induced modification of the innate immune system and activated several signalling pathways related to the immune response (Fig. 6A). The GSEA results demonstrated that decreased UCK2 activated the TNFα/NFκB signalling pathway related SASP (Fig. 6B). Dysregulated genes involved in SASP are listed (Fig. 6C). SASP was a phenotype induced by cell cycle arrest. The secreted SASP factors included CXCL10, which can promote the infiltration of NK cells and T cells. Downregulation of UCK2 increased the expression of ICAM1, which is a downstream adherent molecule of the TNFα/NFκB signalling pathway (Fig. 6D). The immunofluorescence results also showed that knockdown of UCK2 increased the expression of ICAM (Fig. 6E). ICAM1 enhances NK cell cytotoxicity [17]. These results indicate that targeting UCK2 facilitates NK cell surveillance via SASP. Blocking of the TNFα/NFκB signalling pathway did not influence the effect of downregulated UCK2 on the proliferation of HCC cell lines (Fig. 6F). Additionally, gene expression of UCK2 was negatively correlated with NK cell infiltration, particularly the CD56 bright type in HCC but not the NK cell resting and NK cell activation types (Fig. 6G). Moreover, patients with higher levels of activated NK cells and lower levels of UCK2 exhibited a considerably better prognosis (Fig. 6H). These results indicated that targeting UCK2 might sensitize the tumour cells to NK-cell-mediated killing.

### Targeting UCK2 may improve anti-tumour immune response

**Fig. 7:**
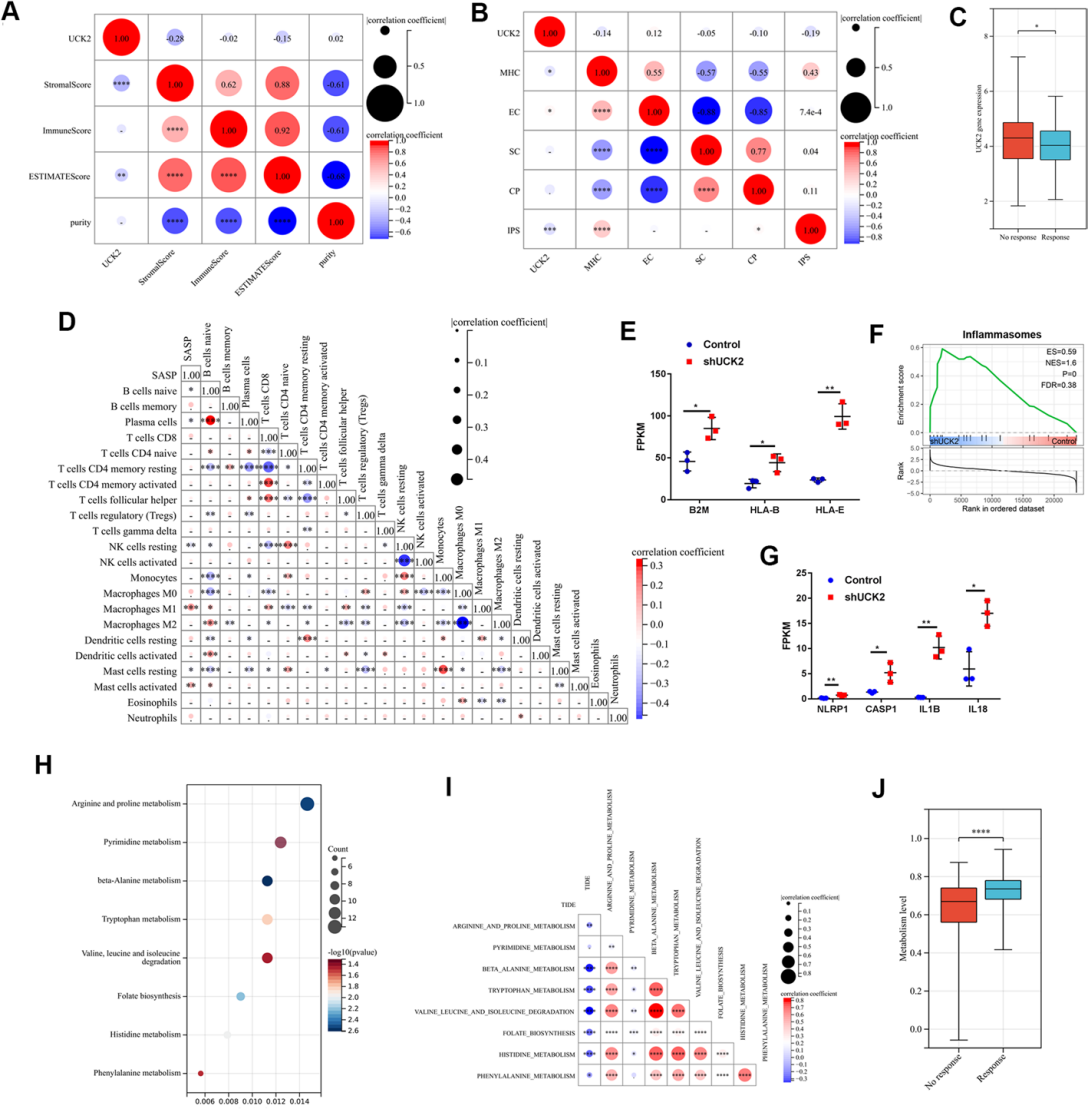
Targeting UCK2 could enhance the sensitive of tumour cell to T-cell-mediated killing. **A** Correlation of UCK2 with ESTIMATE scores and tumour purity. **B** Correlation between UCK2 and IPS scores. **C** Gene expression of UCK2 in immunotherapeutic response versus non-response group from TIDE. **D** Correlation between SASP and immune cell infiltrating derived from CIBFERSORT. **E** Gene expression of B2M, HLA-B and HLA-E in the indicated cell lines. **F** GSEA revealed the level of inflammasomes in the indicated cell lines. **G** Gene expression of NLRP1, CASP1, IL1B and IL18 in the indicated cell lines. **H** Different expression genes enriched in KEGG signalling pathways. **I** Correlation between TIDE score and metabolism related signalling pathways. **J** Downregulated UCK2 induced metabolism altered in immunotherapeutic response versus non-response group from TIDE; *p < 0.05; **p < 0.01; ***p < 0.001; ****p < 0.0001

The ESTIMATE and immunophenoscores (IPS) were determined on the basis of the gene expression of TCGA-LIHC dataset. Elevated UCK2 was only associated with low stromal (Fig. 7A). UCK2 was negatively associated with MHC and IPS but positively associated with the effector cell (EC) score (Fig. 7B). Moreover, patients with higher level of UCK2 predicted to have poorer response to immune checkpoint inhibitors on the basis of TIDE scores (Fig. 7C). These results suggest that patients with high UCK2 levels are less sensitive to immunotherapy. Downregulated UCK2-induced SASP was positively correlated with M1 macrophage infiltration in HCC, promoted anti-tumour immunity and functioned as a biomarker for the immunotherapeutic response (Fig. 7D) [24]. A higher level of UCK2 in HCC was associated with a higher mutation burden (Fig. 3B, Additional file 4: Fig. S4A). Decreased UCK2 elevated the expression of the MHC I molecules, HLA-B, HLA-E and B2M, increasing the chance of neoantigen exposure (Fig. 7E). Additionally, downregulation of UCK2 promoted NLRP1-related inflammasome formation (Fig. 7F, G). MHC I molecules and inflammasome factors are involved in the innate immune system, which instructs the adaptive immune system and sensitizes the tumour to immunotherapy [25]. These data suggest that targeting the UCK2-induced secretory phenotype can improve the immunosuppressive microenvironment. The score for resistance to T-cell-mediated killing was generated based on the TISIDB dataset using the single-sample GSEA method. A high level of UCK2 increased the resistance of tumour cells to T-cell cytotoxicity (Additional file 4: Fig. S4B). Downregulation of UCK2 decreased the expression of genes related to resistance, and these genes were majorly enriched in metabolic pathways (Additional file 4: Fig. S4C). Downregulation of UCK2 regulated several signalling pathways related to metabolism, particularly amino acid metabolism (Fig. 7H). These metabolic pathways were negatively correlated with the TIDE scores (Fig. 7I). The metabolic level based on the above metabolic pathways was generated using the single-sample GSEA method. Thus, patients with higher metabolic levels were predicted to have a better response to immunotherapy (Fig. 7J). These results suggested that targeting UCK2 could also modify the adapt immune response and enhanced the effect of immunotherapy.

## Discussion

Pyrimidine metabolism is critical for tumour progression. UCK is a pyrimidine ribonucleoside kinase that catalyses the first step of the pyrimidine salvage pathway and phosphorylation of several cytotoxic ribonucleoside analogues for cancer treatment [26]. There are two human UCKs: UCK1 and UCK2. UCK1 is ubiquitously expressed in several tissues, whereas UCK2 is expressed in the human placenta and tumour tissues [23]. UCK1 and UCK2 show a sequence similarity of 72%, and UCK2 has a higher catalytic efficiency than UCK1, indicating that UCK2 is an ideal target for tumour treatment [27].

UCK2 has been reported as a prognostic marker for several tumours, including HCC [8]. Several studies have reported that elevated UCK2 promoted HCC proliferation and metastasis [13, 28]. UCK2 upregulated in cancer is partly due to demethylation, which contributes to resistance of cancer to 5-azacytidine treatment [29]. In the present study, we first explore the link between UCK2 and tumour microenvironment. We found that UCK2 was responsive to TGFβ1 stimulation implying the crosslink between microenvironment and tumour cell in the initial stages of tumour formation. The mechanisms of UCK2 promoting the progression of HCC have been reported by several studies. Here, we showed that UCK2 accelerates the cell cycle process through a dual mechanism and identified UCK2 as a novel RNA-binding protein. Downregulation of UCK2 induced cell cycle arrest and activated SASP, which could increase NK cell infiltration and NK-cell-mediated killing. Meanwhile downregulation of UCK2 also causes a secretory phenotype, and MHC I expression which can improve immunosuppression microenvironment. In addition, downregulating UCK2 remodelling metabolism in tumour cells can also increase the response to immunotherapy.

Cancer metabolism has been extensively investigated in recent decades, and metabolic phenotypes adapted by cancer cells can profoundly influence the tumour microenvironment [30]. Because nutrient and oxygen delivery are inefficient owing to poorly differentiated vasculature in tumours, cancer and immune cells compete for nutrients to maintain the demands of rapid proliferation, leading to an anti-tumour defence [31]. Strategies that alter tumour metabolism may improve cancer therapy. However, one limitation of this approach is that critical metabolic pathways are shared by cancer cells and immune cells. For example, the glycolytic pathway, which is essential for dendritic cell survival, is controlled by the mTOR signalling pathway [32]. While aberrant activation of mTOR signalling pathway has been observed in HCC. Targeting mTOR signalling pathway also induces immunosuppression and this effect has been applied in postoperative management of liver transplantation.

UCK2 is mainly expressed in tumour tissues and tumour cells. UCK2 is essential for maintaining the stability of mTOR, and downregulation of UCK2 can specifically inhibit mTOR signalling pathway-related metabolic reprogramming of cancer cells. In addition, targeting UCK2 can relapse amino acid metabolism to decrease the resistance of cancer cells to T-cell-mediated killing [30].

## Conclusions

Targeting UCK2 inhibits HCC progression and improves immune response. This modification could improve the response to immunotherapy in patients with HCC. Therefore, UCK2 is an ideal target for treating HCC to prevent tumour progression and facilitate immunotherapy.

## Supplementary Information

**Additional file 1: Figure S1. **UCK2 was upregulated in HCC and predicted poor prognosis of HCC (A) Gene expression of UCK2 in different datasets, the data was obtained form HCCDB; (B) Kaplan–Meier curves were used to determine the role of UCK2 on the prognosis of HCC, the log-rank test was used to compare survival between different groups.

**Additional file 2: Figure S2. **UCK2 was upregulated by demethylation and TGFβ signalling pathway. (A) The correlation of UCK2 with four DNA methylation transferases in different tumour types; red: DNMT1; blue: DNMT2; green: DNMT3A; purple: DNMT3B; (B) 2 µmol 5-azacytidine-2′-deoxycytidine added in the HCCLM3 and Hep3B cell lines, gene expression of UCK2 was detected by qPCR at different time points; (C) The knockdown efficiency of UCK2 by lentivirus was detected using quantitative real-time PCR; (D) gene expression of SULF2 in the indicated cell lines; (e) Correlation between UCK2 and Treg cell infiltrating.

**Additional file 3: Figure S3. **UCK2 was a novel RNA binding protein. (A) Overview of CHIP peaks targeted by UCK2 over chromosomes; (B) Genes enriched in the signalling pathways; (C) Correlation of UCK2 with PDPK1.

**Additional file 4: Figure S4. **Targeting UCK2 may enhance the sensitivity of tumour cells to T cell-mediated killing. (A) Gene expression of UCK2 with mutation landscape; (B) Correlation of UCK2 with resistant score; (C) Genes involved in the resistant of tumour cells to T cell-mediated killing enriched in signaling pathways.

**Additional file 5: Table S1.** Antibody list.

## Data Availability

The datasets used and analysed during the current study are available from the corresponding author on reasonable request.

## Abbreviations

UCK2: Uridine–cytidine kinase 2
HCC: Hepatocellular carcinoma
SASP: Senescence-associated secretory phenotype
IL1A: Interleukin 1A
TNFα: Tumour necrosis factor α
ICAM1: Intracellular adhesion molecule 1
MHC: Major histocompatibility complex
TCGA-LIHC: The Cancer Genome Atlas Liver Hepatocellular Carcinoma
DMEM: Dulbecco’s modified Eagle medium
FBS: Foetal bovine serum
IP: Immunoprecipitation
IHC: Immunohistochemical
IF: Immunofluorescence
RIP: RNA-binding protein immunoprecipitation
TIDE: Tumour immune dysfunction and exclusion
GSEA: Gene set enrichment analysis
RNAss: RNA expression-based
EREG.EXPss: Epigenetically regulated RNA expression-based
DNAss: DNA methylation-based
DMPss: Differentially methylated probes-based
EREG-METHss: Epigenetically regulated DNA methylation-based
ENHss: Enhancer elements/DNA methylation-based
TMB: Tumour mutation burden
MSI: Microsatellite instability
IPS: Immunophenoscores
EC: Effector cell
