# Supplementary figures and images for: Targeting uridine–cytidine kinase 2 induced cell cycle arrest through dual mechanism and could improve the immune response of hepatocellular carcinoma
Source: Cell Mol Biol Lett. 2022 Nov 26;27:105. doi: 10.1186/s11658-022-00403-y (PMC9707060; doi:10.1186/s11658-022-00403-y)

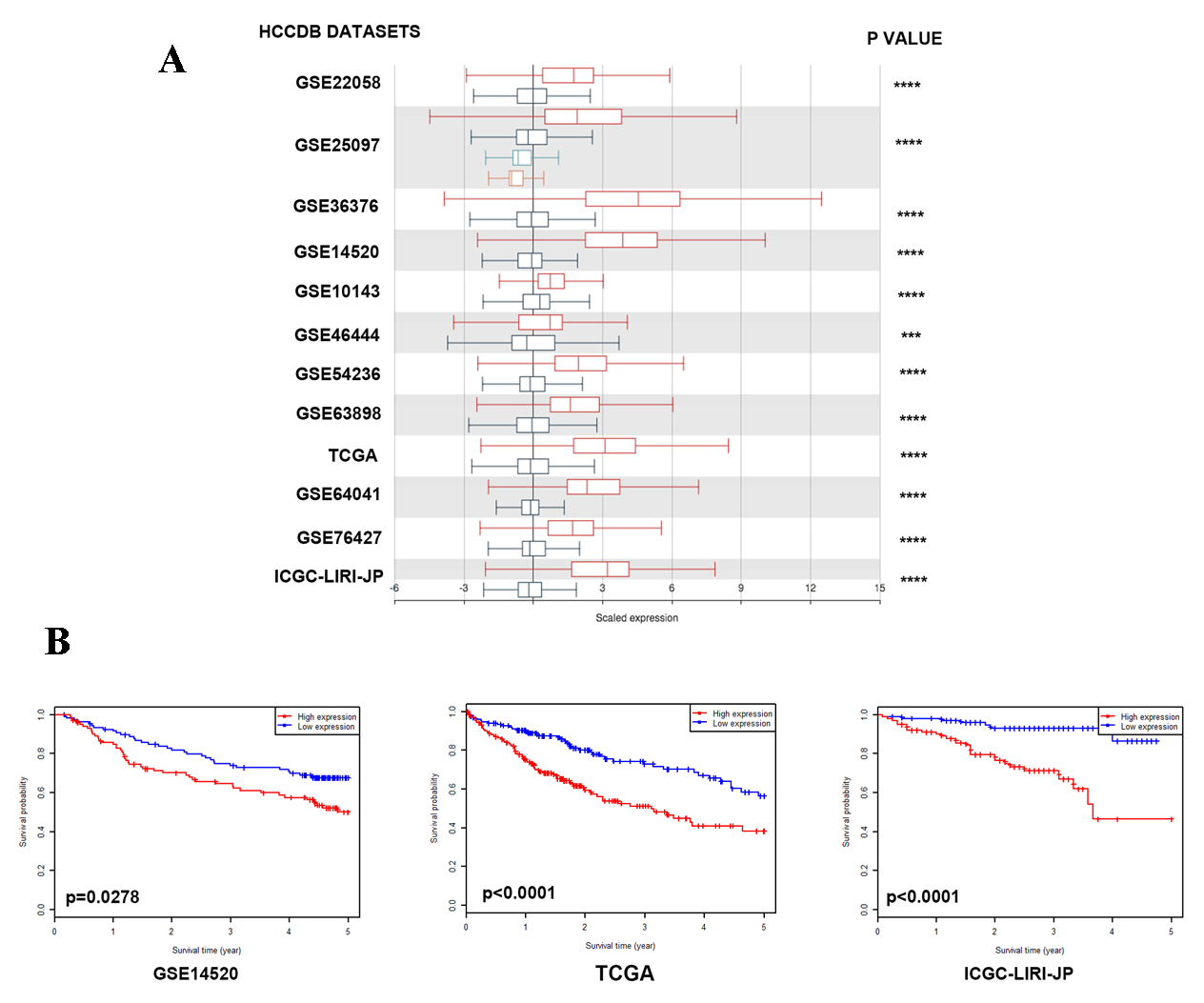

Supplement: Supplementary file 1 — Additional file 1: Figure S1. UCK2 was upregulated in HCC and predicted poor prognosis of HCC (A) Gene expression of UCK2 in different datasets, the data was obtained form HCCDB; (B) Kaplan–Meier curves were used to determine the role of UCK2 on the prognosis of HCC, the log-rank test was used to compare survival between different groups. [file 11658_2022_403_MOESM1_ESM.tif]

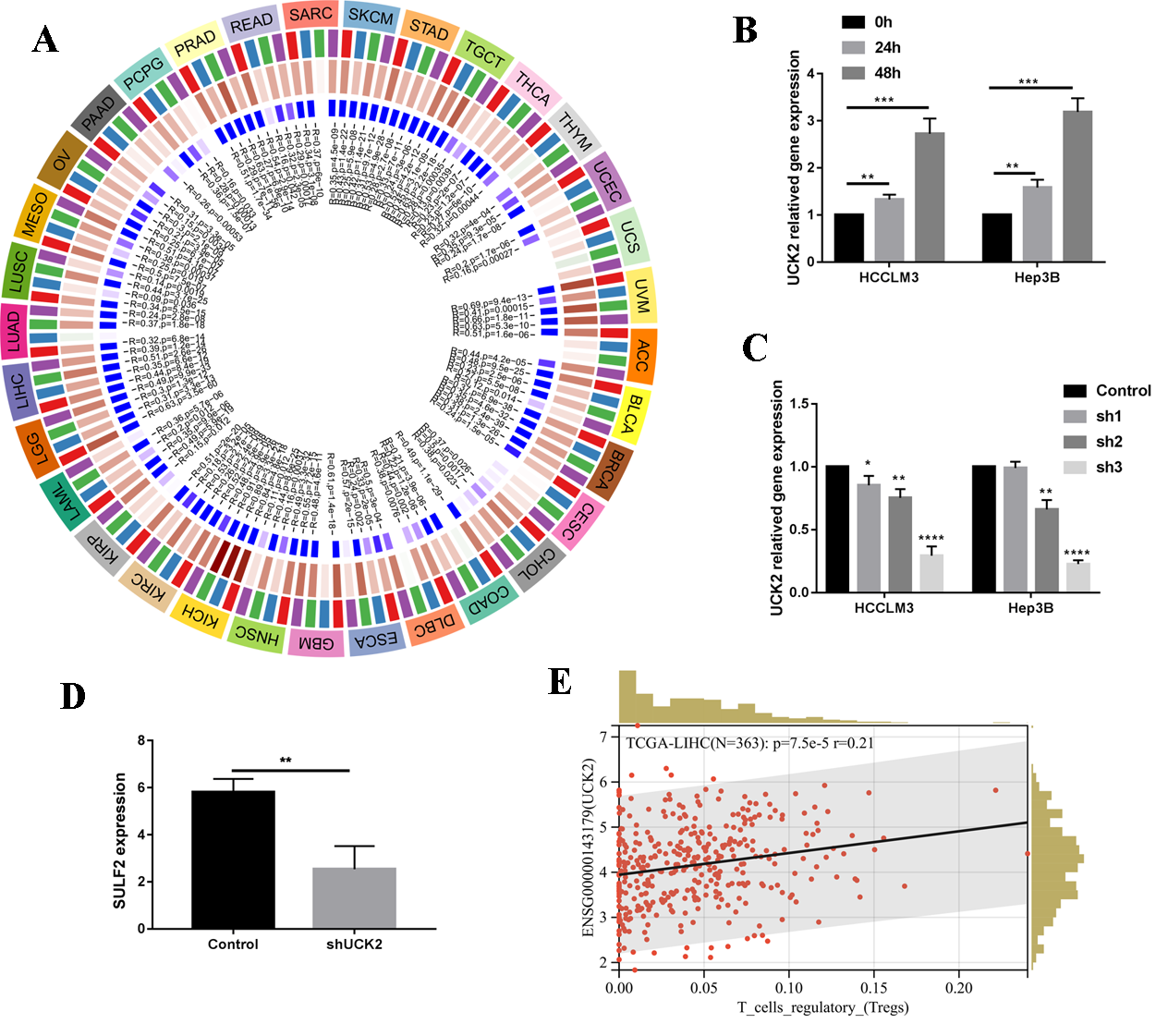

Supplement: Supplementary file 2 — Additional file 2: Figure S2. UCK2 was upregulated by demethylation and TGFβ signalling pathway. (A) The correlation of UCK2 with four DNA methylation transferases in different tumour types; red: DNMT1; blue: DNMT2; green: DNMT3A; purple: DNMT3B; (B) 2 µmol 5-azacytidine-2′-deoxycytidine added in the HCCLM3 and Hep3B cell lines, gene expression of UCK2 was detected by qPCR at different time points; (C) The knockdown efficiency of UCK2 by lentivirus was detected using quantitative real-time PCR; (D) gene expression of SULF2 in the indicated cell lines; (e) Correlation between UCK2 and Treg cell infiltrating. [file 11658_2022_403_MOESM2_ESM.tif]

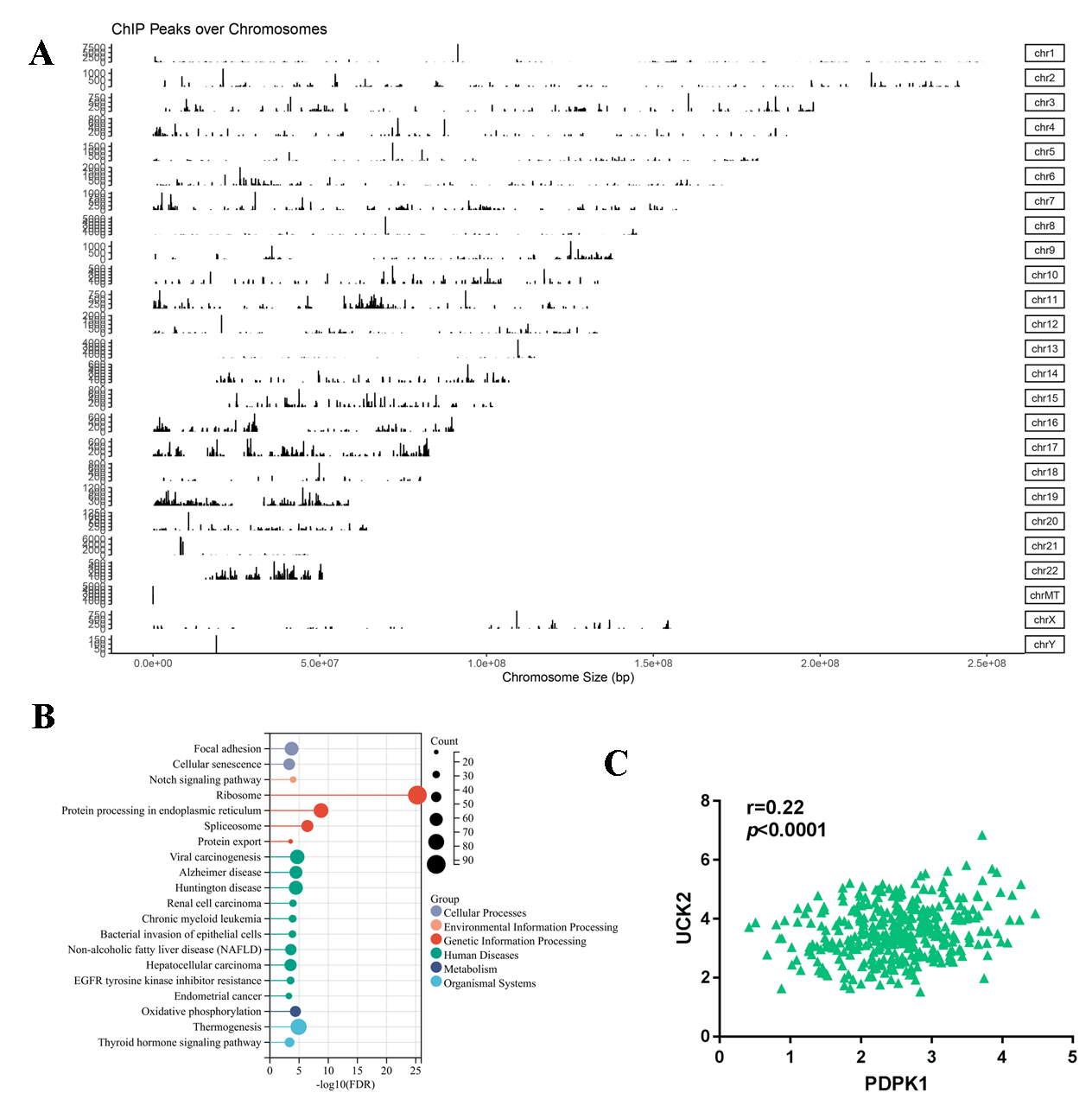

Supplement: Supplementary file 3 — Additional file 3: Figure S3. UCK2 was a novel RNA binding protein. (A) Overview of CHIP peaks targeted by UCK2 over chromosomes; (B) Genes enriched in the signalling pathways; (C) Correlation of UCK2 with PDPK1. [file 11658_2022_403_MOESM3_ESM.tif]

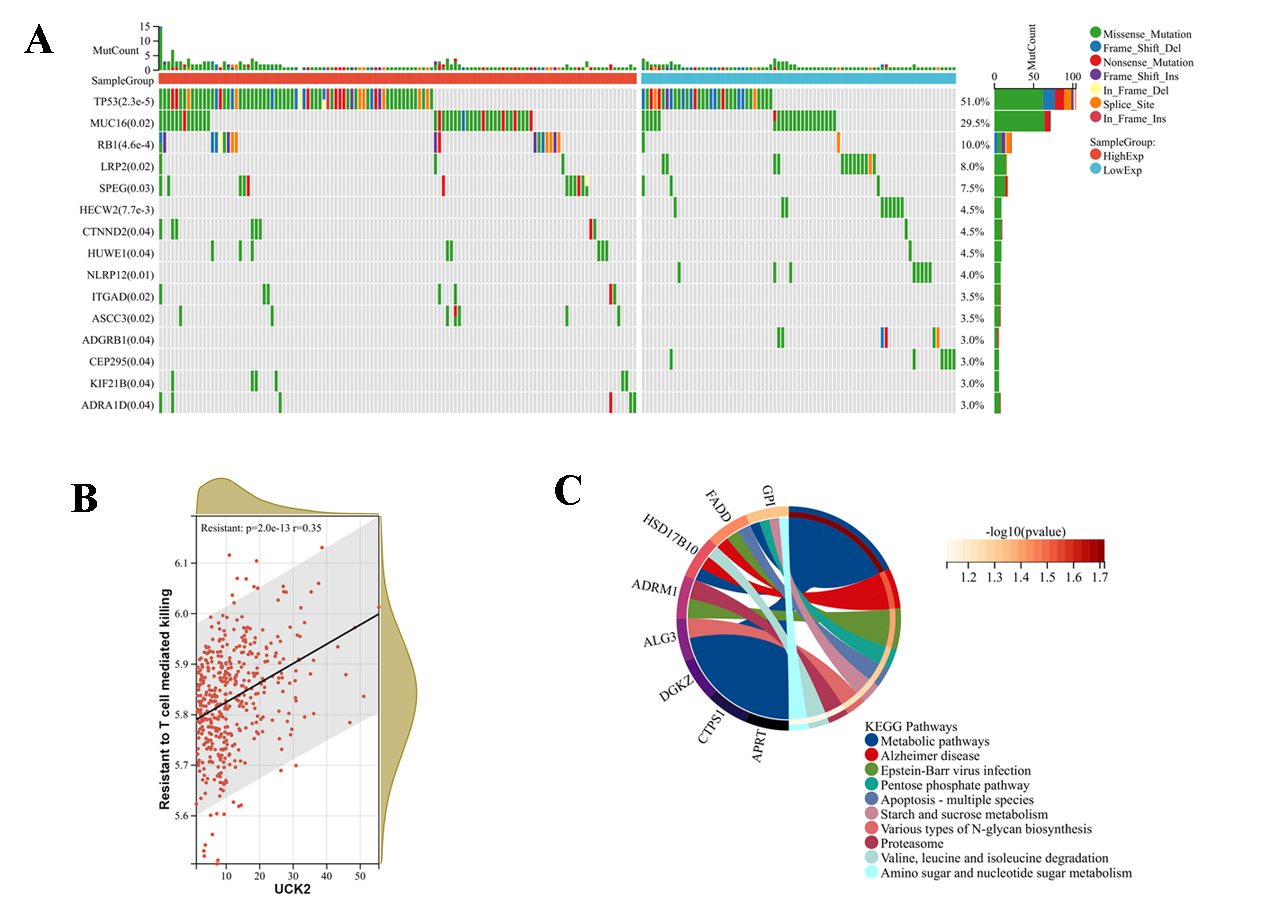

Supplement: Supplementary file 4 — Additional file 4: Figure S4. Targeting UCK2 may enhance the sensitivity of tumour cells to T cell-mediated killing. (A) Gene expression of UCK2 with mutation landscape; (B) Correlation of UCK2 with resistant score; (C) Genes involved in the resistant of tumour cells to T cell-mediated killing enriched in signaling pathways. [file 11658_2022_403_MOESM4_ESM.tif]
