# Supplementary material for: Targeting uridine–cytidine kinase 2 induced cell cycle arrest through dual mechanism and could improve the immune response of hepatocellular carcinoma
Source: Cell Mol Biol Lett. 2022 Nov 26;27:105. doi: 10.1186/s11658-022-00403-y (PMC9707060; doi:10.1186/s11658-022-00403-y)
Supplement: Supplementary file 5 — Additional file 5: Table S1. Antibody list. [file 11658_2022_403_MOESM5_ESM.docx]

| Antibody | Used for | Cat. | Company |
| --- | --- | --- | --- |
| UCK2 | IHC | Ab60222 | abcam |
| UCK2 | Co-IP/RIP | Ab241281 | abcam |
| pmTOR | WB | 5536 | Cell signaling tech |
| mTOR | WB/IHC | 2983 | Cell signaling tech |
| PDPK1 | WB | Ab52893 | abcam |
| pAKT | WB | 4060 | Cell signaling tech |
| AKT | WB | 4685 | Cell signaling tech |
| pNFKB | WB | 3033 | Cell signaling tech |
| NFKB | WB | 8242 | Cell signaling tech |
| ICAM1 | WB/IF | Ab222736 | abcam |
| Secondary antibodies | WB | ZB-2301 (R); ZB-2305 (M) | ZSGB-BIO |
| Secondary antibodies | IF | ZF-0516 (R); ZF-0512 (M) | ZSGB-BIO |
| Secondary antibodies | IHC | Z1203 | Vector Laboratories |

STable1
